# Feasibility of a low-cost magnet tracking device in confirming nasogastric tube placement at point of care, a clinical trial

**DOI:** 10.1038/s41598-024-57455-7

**Published:** 2024-03-25

**Authors:** Hao Li, Kon Voi Tay, Jiajun Liu, Chern Yue Glen Ong, Hau Wei Khoo, Aijin Zhou, Muneaki Miyasaka, Soo Jay Phee

**Affiliations:** 1 Department of Otorhinolaryngology, Tan Tock Seng Hospital, 11 Jalan Tan Tock Seng, Singapore, 308433 Singapore; 2 Department of General Surgery, Woodlands Health, Singapore, Singapore; 3 School of Mechanical and Aerospace Engineering, Nanyang Technological University, Singapore, Singapore; 4 Department of Diagnostic Radiology, Tan Tock Seng Hospital, Singapore, Singapore; 5 Department of Nursing, Tan Tock Seng Hospital, Singapore, Singapore

**Keywords:** Gastroenterology, Medical research, Engineering

## Abstract

An affordable and reliable way of confirming the placement of nasogastric tube (NGT) at point-of-care is an unmet need. Using a novel algorithm and few sensors, we developed a low-cost magnet tracking device and showed its potential to localize the NGT preclinically. Here, we embark on a first-in-human trial. Six male and 4 female patients with NGT from the general ward of an urban hospital were recruited. We used the device to localize the NGT and compared that against chest X-ray (CXR). In 5 patients, with the sensors placed on the sternal angle, the trajectory of the NGT was reproduced by the tracking device. The tracked location of the NGT deviated from CXR by 0.55 to 1.63 cm, and a downward tracking range of 17 to 22 cm from the sternal angle was achieved. Placing the sensors on the xiphisternum, however, resulted in overt discordance between the device’s localization and that on CXR. Short distance between the sternal angle and the xiphisternum, and lower body weight were observed in patients in whom tracking was feasible. Tracking was quick and well tolerated. No adverse event occurred. This device feasibly localized the NGT in 50% of patients when appropriately placed. Further refinement is anticipated.

ClinicalTrials.gov identifier: NCT05204901.

## Introduction

Nasogastric tubes (NGT) are frequently employed to facilitate enteral feeding in patients with dysphagia or to decompress the gastrointestinal tract in patients with ileus or intestinal obstruction. The demand for NGTs is expected to rise alongside the growing incidence of stroke or cancer in aging populations. During NGT insertion, the distal tip of the NGT usually cannot be visualized. This lack of visibility makes it difficult to accurately determine the anatomical location of the tip of the NGT, resulting in a risk of misplacing the tube into the trachea instead of the esophagus. This can cause potentially fatal complications such as aspiration pneumonitis, pneumonia, pneumothorax, in addition to esophageal perforation or failure of bowel decompression^1–3^. Hence, ensuring intragastric placement of NGT is vital both during NGT insertion and before feeding. Currently, various methods are used, such as auscultation of the epigastrium for a “whoosh” while insufflating air, pH testing of the aspirate, ultrasound imaging of the tip of the NGT, electromagnetic (EM) tracking of the NGT, endoscopic visualization of the insertion track by an embedded camera, capnography of gas expired from the lungs into the NGT, and the gold standard of visualizing the NGT on chest X-ray (CXR)^4^.

These methods can be categorized into direct anatomical confirmation (such as CXR, EM tracking^5^, endoscopic visualization^6^, ultrasonography) and indirect physiological confirmation (pH, air insufflation, capnography, and potentially enzymes, impedance, sound or pressure). The existing indirect confirmation methods have several limitations as outlined by Fan et al.^7^. In comparison, direct confirmation methods can be advantageous because they are not affected by the patient's physiological or pathophysiological states except in cases of surgical alteration of the upper gastrointestinal tract or the rare condition of situs-inversus. CXR is considered as the gold standard in terms of accuracy. However, it exposes the patient to ionizing radiation, cannot always be done at the bedside, and takes considerable amount of time to perform, report, and review. This delays feeding or administration of enteral medication, which can be time-essential in some patients. The whole CXR process can also be labour-intensive, costly, and is rarely accessible to patients in the community, let alone private homes where the patients may reside. Moreover, since CXRs are performed after NGT placement, they cannot prevent erroneous placement. In contrast, endoscopic visualization and EM tracking provides a point-of-care test that can confirm correct NGT placement. However, the image quality of endoscopic visualization degrades over time^6^.

The EM tracking system comprises of a magnetic field generator (object to be tracked), a magnetic field receiver, signal processing unit, and a display unit. The magnetic field generator can take the form of either an EM transmitting coil or a permanent magnet. EM tracking has been successfully employed to detect the location of the endoscopic capsule^8–12^ or magnetically actuated capsule^13,14^. In the case of tracking NGT, Sun et al. developed an EM sensing system with 11 magnetic field sensors designed to be worn around the neck^15^. However, its high accuracy (root-mean-square error < 5.3 mm) is valid from – 70 mm to 50 mm from the sensing system along the longitudinal axis, and the tracking accuracy diminishes as the permanent magnet moves further away. Given the average length of the trachea is approximately 100 mm^16^, tracking up to the bronchi with this device becomes challenging. A commercially available EM tracking system, CORTRAK™ EMS-EAS (Avanos Medical, USA)^5^, can enhance the safety and reliability of NGT placement, potentially replacing CXR and reducing time to next clinical procedure^3^. However, it is considered expensive, and requires the patient to use a proprietary NGT which is costly to replace if pulled out (a common scenario).

To reduce the cost of EM tracking, we propose the use of few sensors that can track a permanent magnet moving within the NGT. To achieve this, we first developed a novel tracking algorithm designed to speedily localize a cylindrical magnet in space using only a pair of three-axis magnetic sensors^14,17^. We achieved a high positional tracking accuracy using the proposed algorithm and tracking system in the laboratory, and successfully tracked the insertion of NGT embedded with a permanent magnet at the tip in a mock set-up^17^. Here, we present our clinical prototype with a larger detection range and removable permanent magnet for repeated confirmation of NGT placement at point-of-care. The material cost of our proposed system (including a laptop) is less than 1000 USD, making it relatively cost-effective. Moreover, the magnetic-tipped guidewire is compatible with off-the-shelf 14 Fr Ryle’s tube, a commonly used and affordable NGT. In this report, we present the performance of this prototype in localizing the NGT in the first 10 patients in a hospital setting.

## Material and methods

### Design of the prototype

**Figure 1:**
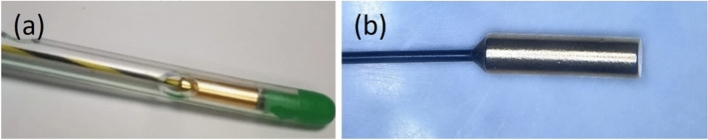
(**a**) The magnet and the guidewire inside a 14Fr Ryle’s tube. (**b**) The microscopic view of the magnet on the guidewire.

**Figure 2:**
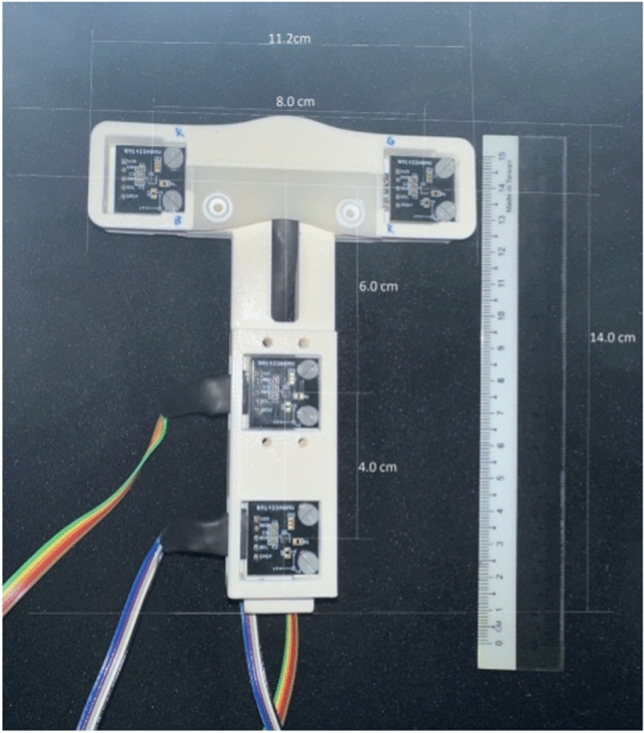
Arrangement of the sensors and the dimensions of the customized 3D printed sensor-housing frame.

**Figure 3:**
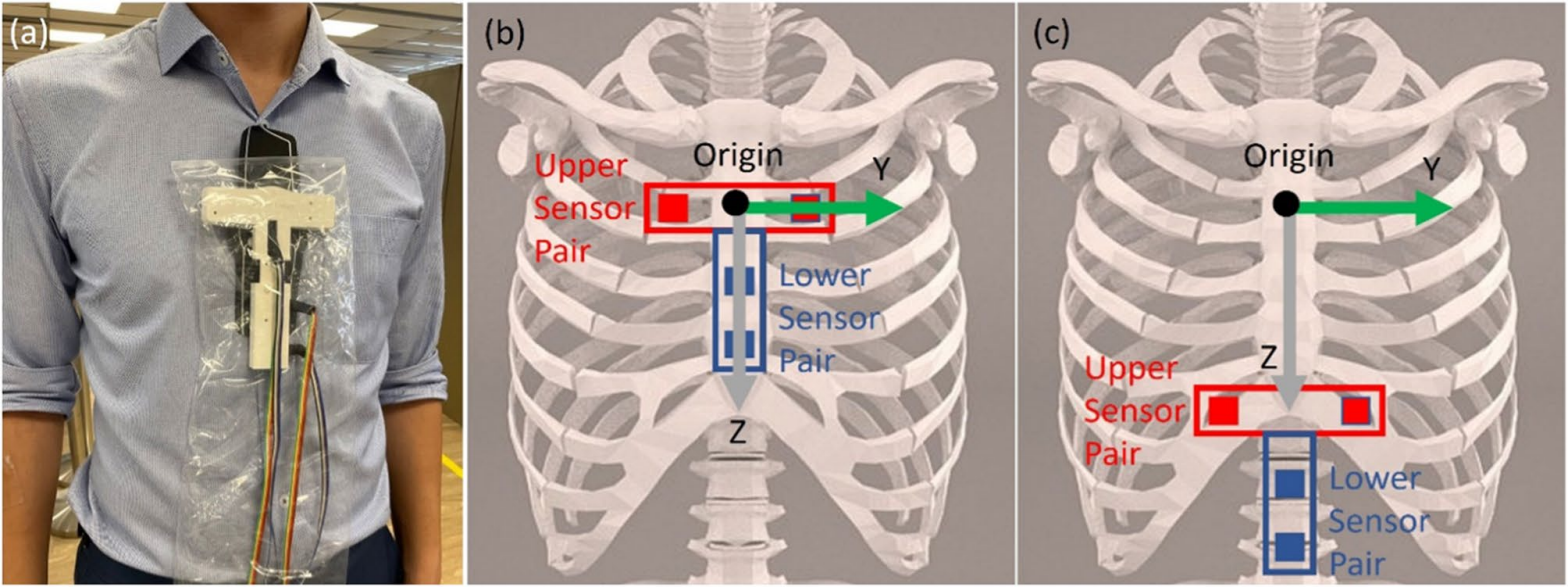
(**a**) Placement of the sensors on the patient; Fig. 3(**b**) and (**c**) Placement of the sensors by surface landmarks. For this study, the upper sensor pair was first placed on the sternal angle as (**b**), then on the xiphisternum as (**c**). In this study, also as illustrated in (**b**) and (**c**), the black dot is the midpoint of the sternal angle and the origin of the coordinates of measurement. Z axis represents the vertical distance from the origin at stern angle, with positive values indicating movement down towards the xiphisternum. Y axis represents horizontal deviation from the vertical reference line, drawn from the intersection between the nasogastric tube or the magnet’s trajectory with the second intercostal space, with positive values indicating deviation to the left and negative values indicating deviation to the right of the sensors.

This NGT localization device consists of a guidewire and a sensor system. To the tip of the guidewire (CE-certified Nitinol with hydrophilic coating, Reborn™ Medical, People’s Republic of China), a D2.8 × 10.5 mm Neodymium N55 grade axially-magnetized gold-coated cylindrical magnet with a D0.95 × 3 mm bottom hole (shichiseitsu.co.jp under Sevenstars Co. Ltd, Japan) is attached using epoxy (EPO-TEK® 353ND, EPOXY TECHNOLOGY, INC, US). The dimension of the magnet is compatible with NGTs 14 Fr or larger, as shown in Fig. 1. The gold coating is preferred to nickel coating because gold prevents erosion by the gastric acid while nickel is a common metal allergen that may trigger systemic allergy^18,19^. The sensor array consists of: (a) four 3-axis magnetic sensors (*BM1422AGMV magnetometer*, Rohm Co. Ltd, Japan); (b) a T-shape housing; (c) an embedded system consisting of *Arduino Uno R3* and *SensorShield-EVK-003* (Rohm Co. Ltd, Japan) to read the signals from the sensors and deliver them to a laptop; (d) a laptop to process, record and display the trajectory of the NGT. The dimension of the sensor array is shown in Fig. 2. The four sensors are grouped into one horizontal upper sensor pair (USP) and one vertical lower sensor pair (LSP). In the first placement, the USP is placed on the sternal angle and aligned with the second intercostal space (a familiar surface landmark of the carina, the origin of the principle bronchi). The LSP is placed straight down the sternum towards the xiphisternum as in Fig. 3a and b. In the second placement, the sensor array is placed at xiphisternum as in Fig. 3c.

### Safety features

The hydrophilic coating of the guidewire ensures smooth insertion into and withdrawal from the NGT. Gold-plating of the magnet prevents its erosion by gastric acid. Neodymium has been used in various medical devices and its serious risk to health appears to be the ingestion of multiple such magnets^20^, which should not occur with our device. Medical grade epoxy is used to join the magnet to the guidewire, and the tensile strength required to detach the magnet from the guidewire is 30 Newton based on our preclinical testing (unpublished data). Prior to the clinical trial, we also inserted this magnetic-tipped guidewire into a 14Fr Ryle’s tube 150 times without observing magnet dislodgement, indicating that it would be safe for clinical use. Moreover, we notified the Health Science Authority of Singapore on the use of this prototype as a Clinical Research Material (reference number CRM2100287) prior to the commencement of the trial.

### Method of confirming intragastric placement

As the magnet is being inserted into the NGT, the location of the magnet is tracked by the sensors and displayed in real-time by the algorithms we previously described^14,17^. At full insertion, the trajectory of the magnet should correspond to that of the NGT. Intragastric placement can be determined by a leftward deviation of the magnet’s trajectory inferior to the xiphisternum because the xiphisternum is a landmark for the cardia of the stomach, and the stomach lies in the left hypochondrium except in the rare case of situs inversus, or previous gastroesophageal surgery (can be known prior to the insertion of NGT). Lateral deviation superior to the xiphisternum coupled with the inability to advance the magnet inferior to the transverse plane of the xiphisternum indicates erroneous NGT placement into the lungs. To indicate the level of the xiphisternum on the tracking display, we can measure the distance from the midpoint of the sternal angle to the tip of xiphisternum on the patient and input that into the tracking display. (The method of computing the location of the NGT are provided in Appendix A in *Supplementary Materials*).

### Trial design

This is a single-arm study conducted in an urban, tertiary general hospital in Singapore (Tan Tock Seng Hospital). In the morning of every weekday, the radiologists screened the chest X-rays done for the confirmation of NGT placement in the last 24 h to determine the eligibility for recruitment based on the criteria below. The radiological eligibility is designed to ensure that the NGT placement with respect to the sternal angle and Z-axis (pointing down towards the xiphisternum) can be measured from the chest X-ray. Next, the research assistant and the clinicians screened the patients who met radiological eligibility to determine their clinical eligibility which is based on the principle of minimal necessary risk. Dually eligible participants were approached for informed consent. Subsequently, the trial intervention was performed in one setting at the bedside within 10 days of the chest X-ray. Follow-up was required if an adverse event occurred. The participants were reimbursed with a small amount of cash at the end of the procedure.

### Eligibility

#### Inclusion criteria

- Patients from the general ward who have an NGT inserted within the last 10 days and chest X-ray confirming its correct placement.
- The NGT is a Ryle’s tube, size 14 Fr or larger.
- Negative for COVID-19 or not infective.
- Age ≥ 21 years.
- Body-mass-index < 35 kg/m^2^.
- Height < 1.9 m.
- Mentally competent for informed consent.

#### Exclusion criteria

- Radiological.
  - On chest X-ray, the length of the NGT distal to the gastroesophageal junction is less than 12 cm.
  - The NGT is kinked within 10 cm of its tip.
  - The chest X-ray is rotated.
  - The NGT cannot be visualized in the mediastinum.
  - The second intercostal space cannot be visualized on the X-ray.
- Vital signs.
  - Heart rate ≥ 100 or < 60 beats/min.
  - Systolic blood pressure ≥ 160 or < 100 mmHg.
  - SpO_2_ < 92% in patients with chronic lung disease or < 95% in patients without chronic lung disease.
  - Temperature ≥ 38 °C.
- Pectus carinatum or excavatum.
- Patients with the following implants.
  - Pacemaker.
  - Automated cardioverter defibrillator.
  - Ferromagnetic coronary stents, heart valves, implants, surgical clips of the head, neck, thorax, abdomen, or pelvis.
- Following conditions within the last 30 days.
  - Upper gastrointestinal bleeding.
  - Oesophageal or gastric surgery.
  - Stroke.
  - Myocardial infarction.
  - Aortic dissection.
  - Ruptured aortic aneurysm.
- Allergy to neodymium, gold, epoxy, or nitinol.
- Not able to understand English, Chinese, Malay, or Tamil.
- Women whose last menstrual period commenced more than 4 weeks before recruitment, unless they have a negative pregnancy test.

### Performance of chest X-ray

Chest X-rays were performed either in the radiology department using an overhead system (Fujifilm Visionary Suite) or at the bedside using a portable X-ray machine (Fujifilm FDR Go PLUS). For NGT localization, radiographs were acquired in anteroposterior (AP) projection, that is, the X-ray source projects from the front of the patient onto a flat panel placed behind (or underneath) the patient. Our routine exposure settings for chest X-ray are 75 kilovoltage peak (kVp) and 3.2 milliampere-seconds (mAs), though technologists may tweak them according to the patient’s body habitus. Following X-ray acquisition, the technologist screens the image for quality before uploading them for reporting by the radiologist.

### Trial intervention

**Figure 4:**
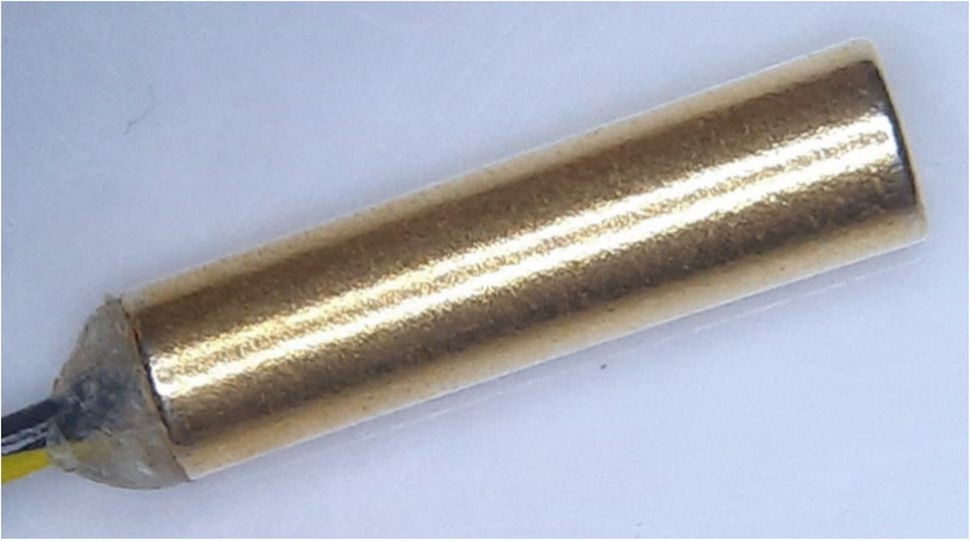
Inspection of the magnet under the microscope showing no evidence of erosion by gastric content.

The patients were allowed to recline in bed or sit in a chair. We first measured on them (a) the distance from the midpoint of the sternal angle to the tip of the xiphisternum; (b) the vertical distance from the inferior border of the head of clavicle to the second intercostal space; (c) the circumferences of the chest or abdomen at the plane of the second intercostal space, the tip of the xiphisternum, and the umbilicus, respectively. Next, the sensors, covered in a sterile plastic sleeve, were clicked onto a pad. The pad was stuck on the sternum of the patient with the upper sensor pair placed centrally across the sternal angle (Micropore™ were used in some patients to further secure the sensors to the chest). To prevent the magnet from extruding from the NGT, we did not insert it into the last 10 cm of the Ryle’s tube where side holes are present. To ensure this, a stopper was locked on the guidewire at 95 cm from the tip of the magnet because the Ryle’s tubes measure 105 cm in length. After these preparations, the sensor system was initialized, during which the environmental magnetic flux density was measured without the magnet’s presence and assumed to be constant subsequently. After initialization, the clinicians rinsed the NGT with a small amount of saline, disinfected the guidewire and magnet with ethanol wipes, and began inserting them into the NGT. After the clinician felt a loss of resistance (a “give”) when the NGT went past the nasopharynx, tracking was started. The tracking was not started in the nose because the tracking algorithm assumes that the magnet is vertical. Thus, the “give” indicates that the magnet has begun its descent into the pharynx, which is approximately vertical. We inserted the magnet into the NGT at a speed similar to that experienced in clinical practice. Two insertions were to be performed per patient, once with the upper sensor pair placed on the sternal angle and another with it placed on the xiphisternum. After insertion, the magnet was immediately withdrawn, and its trajectory was tracked during withdrawal. After the withdrawal was complete, the sensors were removed from the patient. Pain score and the site of discomfort were assessed by enquiring the patient. Finally, the magnet and guidewire were disinfected with ethanol wipes, taken to the laboratory, and examined under the microscope for any visible erosion as shown in Fig. 4.

### Outcomes measured

The primary outcome is the concordance between the trajectory of the NGT visualized by magnetic tracking and by chest X-ray. This is measured by superimposing these trajectories onto each other starting from the same origin centred on the sternal angle, i.e. the intersection between the level of the second intercostal space and either the NGT on chest X-ray or the magnet’s trajectory on the tracking output. From this common origin, a global coordinate system is deployed with the Z-axis pointing down towards the xiphisternum and the Y-axis pointing towards the patient's left, as shown in Fig. 3b. Distances on the Y-axis from both the chest X-ray and the tracking result are then measured at 2 cm intervals along the Z-axis and compared, starting from the sternal angle and ending at the lower limit of tracking (see Appendix A in *Supplementary Materials* for more details on data processing). The secondary outcomes are (a) the feasibility of detecting a leftward deviation of the NGT inferior to the xiphisternum; (b) the test–retest reliability comparing insertion to withdrawal; (c) the time taken to complete the tracking; (d) the level of discomfort experienced by the patient; (e) the body habitus in which tracking is likely feasible. The safety parameters measured include detachment of the magnet from the guidewire, erosion of the magnet, and dermatitis at the site of sensor placement. Clinical data concerning the indication of NGT insertion, demographics and the patient’s body-mass-index were also collected. Finally, the agreement between the upper and lower sensor pairs about the location of the magnet is calculated to further understand the performance of the device (see Appendix B in *Supplementary Materials* for the method of calculation).

### Sample size

As this is a feasibility study, we followed Julious’ recommendation of 12 subjects^21^. We aimed to recruit 6 male and 6 female patients, but recruitment stalled after 10 patients completed the study because of a new surge in COVID-19. Therefore, an unplanned interim analysis was performed and showed that the data was sufficient in determining feasibility of the device in localizing NGT.

### Statistical methods

As the sample size is small, for continuous variables, both mean, median, their standard deviation and range are provided. Proportions are calculated for categorical variables. These are performed in STATA (Version 17.0, Basic Edition, TX, USA).

### Ethics approval and monitoring

This trial is performed in accordance with the Declaration of Helsinki, registered on ClinicalTrials.gov on 24/01/2022 (identifier NCT05204901), approved by both the Domain Specific Review Board of the National Healthcare Group of Singapore (reference number 2021/00435) and the Institutional Review Board of Nanyang Technological University (Reference number: IRB-2021-959). The trial was monitored by an independent auditor. All participants provided written informed consent.

## Results

### Characteristics of the participants

**Figure 5:**
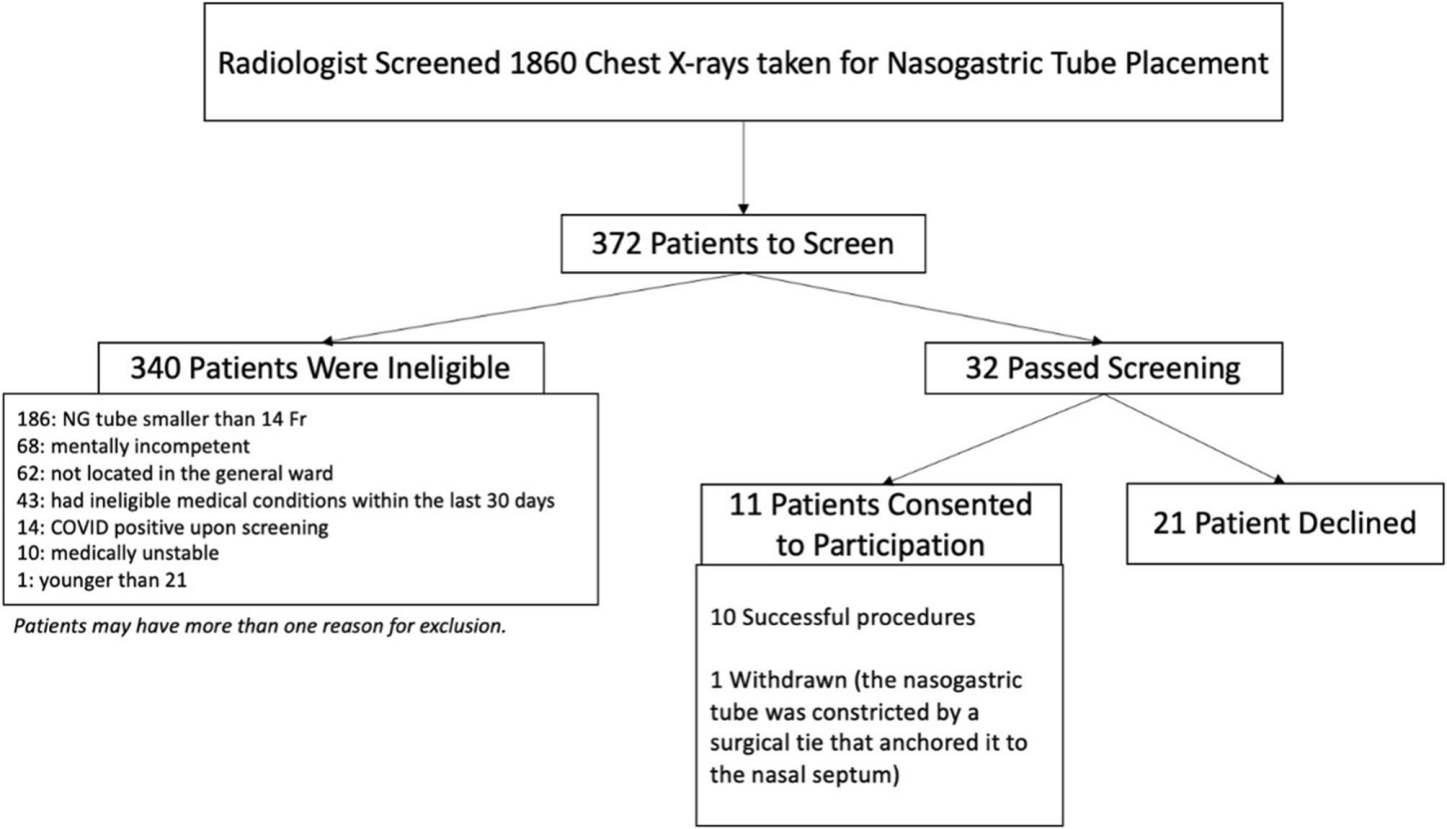
Recruitment flowchart.

From 4 May to 7 December 2022, a total of 1860 chest X-rays (CXR) taken after NGT insertion were screened, 1488 were excluded by radiological criteria or due to repeated X-rays taken from the same patient, 340 were excluded by clinical criteria. Eleven out of the remaining 32 patients consented to the trial, but one withdrew because the NGT was unexpectedly constricted by a surgical stitch that prevented the insertion of the magnet. The remaining 10 patients (6 males and 4 females) completed the trial (see Fig. 5 for a flowchart of the recruitment process). Their characteristics and body dimensions are presented in Table 1. The median time interval between CXR and the trial intervention was 1.5 days (range 0 to 4 days). The size of the Ryle’s tube was 14Fr in 7 patients and 16Fr in the other 3. Placement of the sensors on sternal angle was achieved in all 10 patients, but placement on the xiphisternum was aborted in 2 patients because of unexpected power outage of the tracking computer in one and concern about the comfort of the patient in the other.

**Table 1:** Characteristics of the participants.

	No. = 10
Age (years)	Mean 59 (SD 13.1), median 60.5 (range 25.0–73.0)
Sex	6 male, 4 female
Height (m)	Mean 1.62 (SD 0.06), median 1.61 (range 1.50–1.70)
Weight (kg)	Mean 60.15 (SD 8.16), median 60.75 (range 47.0–75.0)
Body-mass index (kg/m^2^)	Mean 22.90 (SD 2.38), median 23.10 (range 18.36–27.55)
Chest circumference at 2nd intercostal space (cm)	Mean 86.5 (SD 10.8), median 89.5 (range 65.0–98.5)
Chest circumference at xiphisternum (cm)	Mean 82.7 (SD 96.3), median 80.4 (range 71.0–95.5)
Abdominal circumference at umbilicus (cm)	Mean 77.9 (SD 17.8), median 81.8 (range 36.0–94.5)
Vertical distance between the inferior border of the head of the clavicle and the 2nd intercostal space (cm)	Mean 7.5 (SD 2.3), median 6.8 (range 5.5–12.5)
Vertical distance between the sternal angle and xiphisternum (cm)	Mean 12.8 (SD 2.62), median 13.3 (range 8.5–16.5)
Time interval between the chest X-ray and the insertion of the magnet (day)	Mean 1.5 (SD 1.2), median 1.5 (range 0–4)
Indication for nasogastric tube insertion	bowel obstruction in 3 patients, partial gastric outlet obstruction, sigmoid volvulus, colon carcinoma, appendicectomy, peritonsillar abscess, base of tongue carcinoma and facial swelling from trauma in the other 7 patients, respectively

*SD* standard deviation.

### Patient tolerance

Full insertion of the magnet-tipped guidewire until the 95 cm mark was achieved in all cases. Insertion, withdrawal, and the tracking of the magnet took an average of 94.3 s for the first attempt and 91.7 s for the second. Three patients experienced pain, with a pain score of 3 out of 10 in 2 patients and 1 out of 10 in the other, localized to the nose in 2 patients and to the throat in 1. Five minutes after withdrawal of the magnet, only 1 patient continued to experience pain (in the nose) with a score of 1 out of 10. No adverse event was encountered (Table 2).

**Table 2:** Clinical and safety outcomes.

	No. = 10
Duration of 1st insertion (seconds)	Mean 94.3 (SD 24.8), median 94 (range 59–131)
Duration of 2nd insertion (seconds)	Mean 91.7 (SD 32.0), median 85.5 (range 41–140)
Highest pain score (from 0 to 10) during the insertion or withdrawal of the magnet	3 in 2 patients 1 in 1 patient 0 in 7 patients
Highest pain score (from 0 to 10) 5 min after completion of the trial interventions	1 in 1 patient 0 in 9 patients
Location of the pain	Nose in 2 patients, throat in 1 patient
Detachment of the magnet	None
Erosion of the magnet	None
Dermatitis at the sensor placement site	None

### Performance of the tracking device

**Figure 6:**
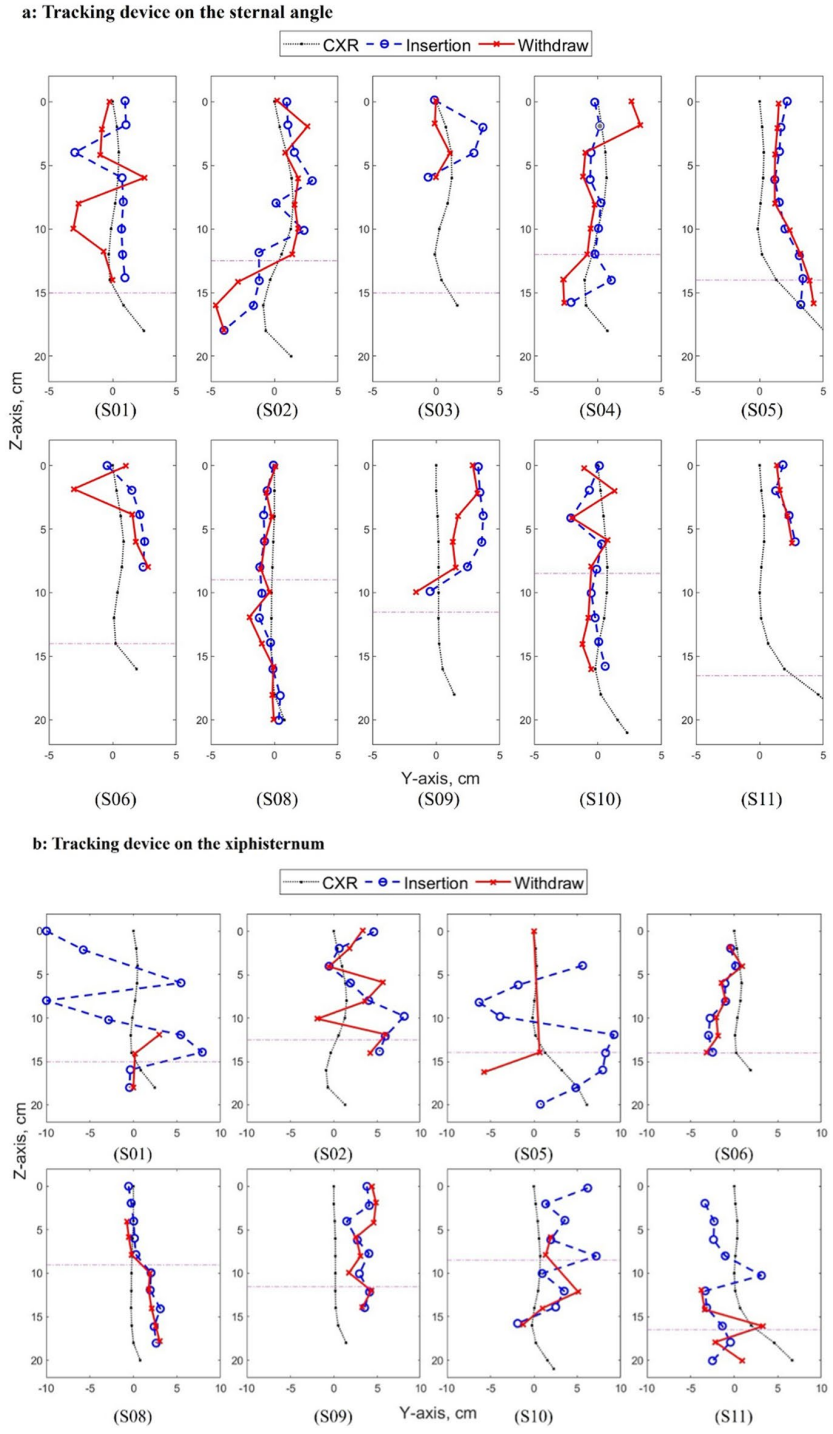
Location of the nasogastric tube determined by magnet tracking versus chest X-ray (CXR) for (**a**) tracking device was placed on the sternal angle, and (**b**) tracking device was placed on the xiphisternum. Each sub-graph shows the data from an individual labelled by the subject (S) number. The processed tracking data for insertion are indicated by blue dash line with circle marker. The processed tracking data for withdrawal are indicated by red line with cross marker. The black dot line with dot markers are the measurements from CXR. The dot-dash horizontal purple line indicates the level of the xiphisternum in each patient. The data points with markers are spaced 2 cm apart in Z axis to match with the CXR measurements. The coordinate system has been introduced in Fig. 3.

Figure 6 compares the localization of NGT by the tracking device against that by chest X-ray (CXR). With respect to the primary outcome, tracking corresponded to CXR more closely when the upper sensor pair (USP) was placed on the sternal angle than when it was placed on the xiphisternum (see Table 3 for the exact difference). Placing the USP on the xiphisternum resulted in overt discordance. However, when the USP was placed on the sternal angle, tracking discontinued prematurely in subjects 03, 06, 11, and did not reach the xiphisternum in subjects 01 and 09. In the remaining subjects (02, 04, 05, 08, 10), the tracked location of the NGT deviated from that of CXR by an average of 0.55 to 1.63cm, and a downward tracking range of 17 to 22cm from the sternal angle was achieved (calculated from Table 3). The NGT turned solely to the left inferior to the xiphisternum in 7 out of the 10 patients but turned to the right before turning to the left in the other 3. The tracking result corresponded to the initial turn of the NGT inferior to the xiphisternum in 5 out of 10 patients (subjects 02, 04, 05, 08, 10) when the USP was placed on the sternal angle, but only in 1 patient when it was placed on the xiphisternum (subject 08). The test–retest variability, measured by the average difference in the magnet’s location between insertion and withdrawal, is improved in all but subject 06 when the USP was placed on the sternal angle compared to the xiphisternum (Table 3).

**Table 3:** Average difference between the location of the nasogastric tube determined by magnet tracking and by chest X-ray.

	Sub 01	Sub 02	Sub 03	Sub 04	Sub 05	Sub 06	Sub 08	Sub 09	Sub 10	Sub 11
(a) upper sensor pair on the sternal angle										
${\mu }_{y,ic}$	1.15	1.29	1.67	0.73	1.60	1.29	0.54	2.78	0.83	1.84
${\mu }_{y,wc}$	1.43	1.40	0.56	1.58	1.65	1.67	0.56	1.99	1.12	1.70
${\mu }_{y,iw}$	2.08	1.33	1.59	1.46	0.40	1.54	0.38	1.16	0.89	0.30
${n}_{ic}$	8	10	4	9	9	5	11	6	9	4
${n}_{wc}$	8	10	4	9	9	5	11	6	8	4

	Sub 01	Sub 02	Sub 05	Sub 06	Sub 08	Sub 09	Sub 10	Sub 11		
(b) upper sensor pair on the xiphisternum										
${\mu }_{y,ic}$	5.79	3.41	4.87	1.96	1.39	3.22	2.85	3.80		
${\mu }_{y,wc}$	2.06	3.23	3.20	1.84	1.60	3.49	1.71	4.36		
${\mu }_{y,iw}$	3.55	2.23	10.64	0.53	0.43	0.91	1.92	2.10		
${n}_{ic}$	9	8	9	7	11	8	9	10		
${n}_{wc}$	3	8	3	7	9	8	5	5		

Unit of measurement: cm.

Sub: research participant.

${\upmu }_{{\text{y}},{\text{ic}}}$ *:* average absolute distance in Y-axis between the tracking result and the chest X-ray during insertion of the magnet.

${\mu }_{y,wc}$: average absolute difference in Y-axis between the tracking result and chest X-ray during withdrawal of the magnet.

${\mu }_{y,iw}$: average absolute difference in Y-axis between the tracking results of insertion and withdrawal of the magnet.

${{\text{n}}}_{{\text{ic}}}$: number of comparisons made during insertion of the magnet (spaced 2 cm apart vertically).

${{\text{n}}}_{{\text{wc}}}$: number of comparisons made during withdrawal of the magnet (spaced 2 cm apart vertically).

### Agreement between the upper and lower sensor pairs about the location of the magnet

**Figure 7:**
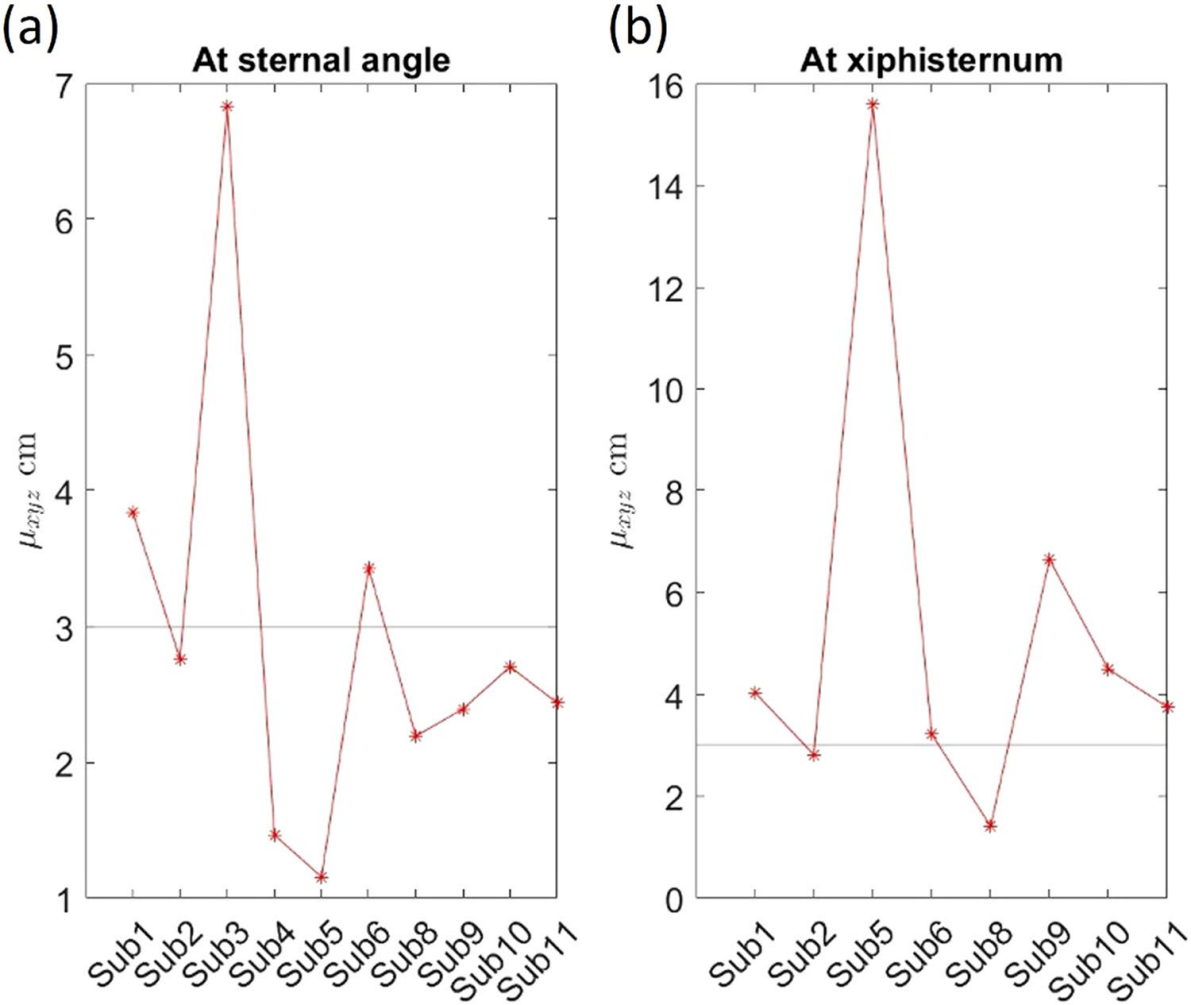
Agreement between the upper and lower sensor pairs about the magnet’s location. The y-axis of the plots is the value of ${\mu }_{xyz}$, which is the average absolute difference in the measured location of the magnet between the upper and lower sensor pairs. The Sub refers to the research participant.

To further assess if the tracking is robust, the magnet’s location can be taken as the ground truth instead of the NGT’s location on X-ray, and both the upper and lower sensor pairs should agree on the location of the magnet within a narrow range. Figure 7 shows the average agreement between the USP and LSP about the magnet’s location in each subject. This was within 3 cm in 6 out of 10 subjects when the USP was on the sternal angle, but in 2 out of 8 subjects when the USP was on the xiphisternum. Agreement within 2 cm was observed in 2 out of 10 subjects when the USP was on the sternal angle, and in 1 out of 8 subjects when the USP was on the xiphisternum.

### Clinical factors associated with the feasibility of localizing the nasogastric tube by the tracking device

**Figure 8:**
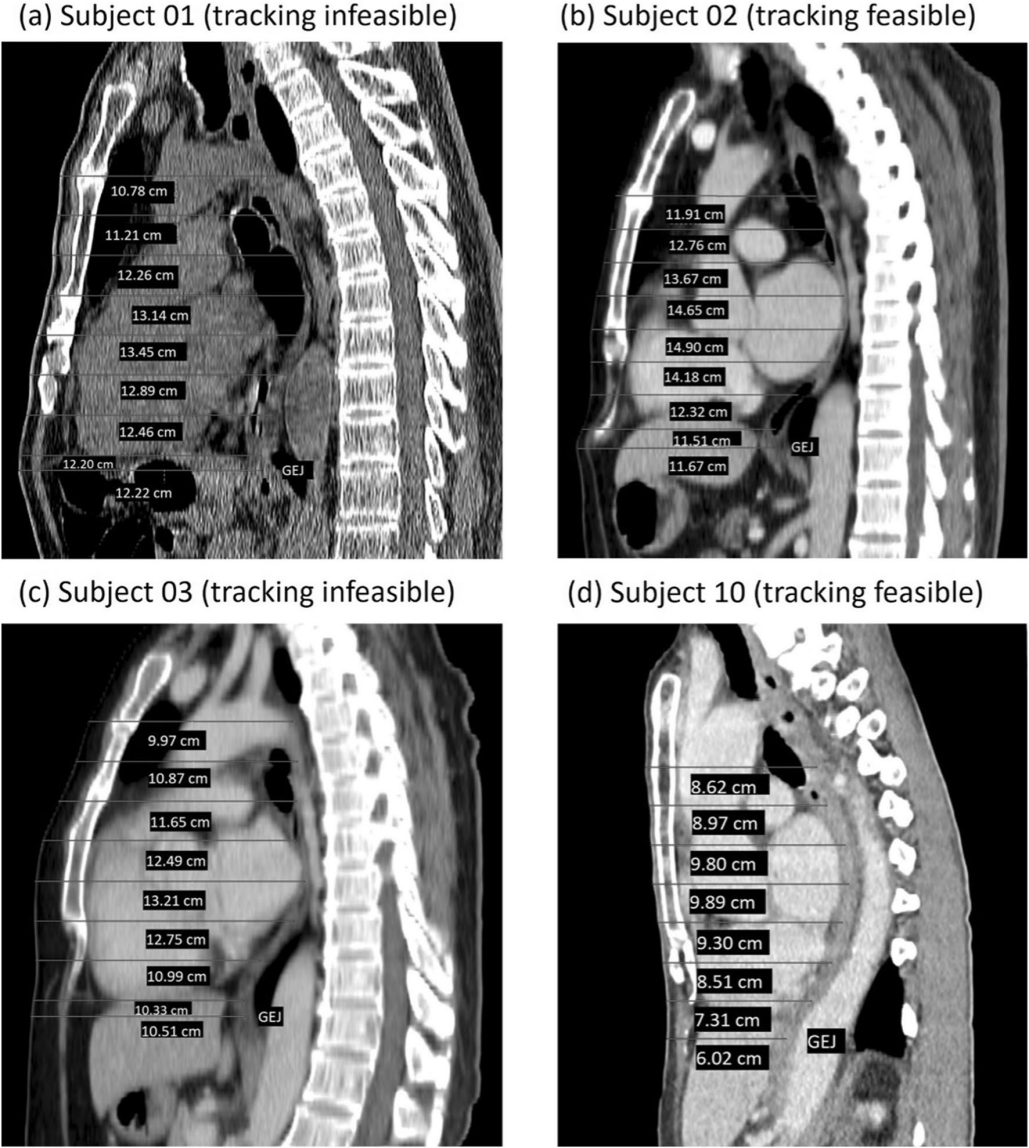
Distance between the esophagus and anterior chest wall in participants who had computed tomography before the trial. *GEJ* gastroesophageal junction.

Localization of the NGT is considered feasible in subjects 02, 04, 05, 08 and 10 because the turn of the NGT inferior to the xiphisternum could be correctly visualized by the device when the USP was placed on the sternal angle. Thus, the clinical characteristics of these 5 patients are compared with those of the other 5 in whom tracking was infeasible (Table 4). No statistical analysis is performed because of the small sample size. Patients in whom tracking was feasible appear to have a shorter vertical distance between the sternal angle and the tip of the xiphisternum, lower weight, lower body-mass-index, and fewer male than patients in whom tracking is infeasible, but height, chest or abdominal circumferences do not appear to differ. In 4 patients, the distance between the lumen of the esophagus and the anterior chest wall could also be measured because computed tomography of the thorax was performed within a year before the trial, but this distance does not appear to differ between those in whom tracking was feasible and was not (Fig. 8).

**Table 4:** Clinical factors associated with the feasibility of localizing the nasogastric tube by the magnet tracking device.

	Feasible: N = 5	Not feasible: N = 5
Age (year)	55.2 (SD 17.31) Median: 60 (range 25–67)	62.8 (SD 7.22) Median: 61 (range 55–73)
Gender (% male)	40%	80%
Height (m)	1.61 (SD 0.07) Median 1.6 (range 1.5–1.7)	1.63 (SD 0.05) Median 1.62 (range 1.58–1.7)
Weight (kg)	56 (SD 7.42) Median 55 (range 47–64)	64.3 (SD 7.19) Median 61 (range 57–75)
Body-mass-index (kg/m^2^)	21.62 (SD 2.03) Median 21.80 (range 18.36–23.80)	23.17 (SD 2.13) Median 23.63 (range 21.72–27.55)
Chest circumference (cm)		
2nd intercostal space	84.8 (SD 8.76) Median 840 (range 740–970)	88.18 (SD 13.40) Median 914 (range 650–985)
Xiphisternum	78.7 (SD 9.67) Median 76.0 (range 71–95.5)	86.66 (SD 8.70) Median 90.5 (range 73–95.5)
Umbilicus	80.0 (SD 9.87) Median 80.5 (range 69 –94.5)	75.8 (24.63) Median 90.5 (range36–93)
Vertical distance (cm)		
From clavicle to 2nd intercostal space (sternal angle)	7.4 (SD 1.88) Median 7.5 (range 5.5–9.5)	7.6 (SD 2.90) Median 6.0 (range 5.5–12.5)
From 2nd intercostal space (sternal angle) to xiphisternum	11.2 (SD 2.36) Median 12.0 (range 8.5–14.0)	14.4 (SD 1.85) Median 15.0 (range 11.5–16.5)

## Discussion

This is a first-in-human trial of a low-cost magnet tracking device for the confirmation of NGT placement. Because abundant caution was placed on safety, we did not insert the magnet until the end of the Ryle’s tubes to prevent extrusion of the magnet through the side-holes. This increased the difficulty in recruitment as we still needed to visualise the left turn of the NGT for the confirmation of gastric placement. Therefore, only patients whose NGTs were inserted deep into the stomach were eligible. Moreover, only Ryle’s tubes were eligible because they have a closed end that further prevents extrusion of the magnet, but patients outside the surgical service tend to have different brands of NGT that have an open end. In addition, those patients tend to have NGTs thinner than 14Fr, and tend to lack the mental capacity to consent. This explains the high exclusion rate in the selection of participants for this trial.

The patients recruited are Asians who have a wide range of body dimensions especially with respect to abdominal circumference. Majority of the patients were comfortable during the magnet tracking, which took less than 2 min in most instances. The mild pain in the nose or throat experienced by some patients can be due to the resistance encountered when inserting the magnet past the nasopharynx. Moistening the guidewire before insertion can reduce the resistance. Once past the nasopharynx, the magnetic-tipped guidewire was inserted or withdrawn from the NGT without much resistance. Thus, no magnet dislodgement or wire breakage occurred.

To minimize the risk of ionizing radiation to the participants, we did not subject them to fluoroscopy that could confirm the location of the NGT in real-time as the magnet was inserted or withdrawn. Instead, we used the X-ray taken a few days earlier as a surrogate of the NGT’s location at the time of the trial. Theoretically, the NGT could have moved in the time interval, but in practice, nurses in our hospital routinely mark the length of NGT inserted into the patient by placing a tape on it at the level of the nostril. Moreover, they have to ensure that the tube remains at this marked position every shift and prior to every feeding. If the NGT had shifted after the X-ray had confirmed its location, our nurses would have readjusted the tube to the marked position. Failure to do so would result in the removal of the NGT, reinsertion and typically another X-ray, documented in the records. Therefore, at the time of the trial, the NGT should have remained in the location visualized on the last X-ray. That we completed the trial within 4 days of the X-ray further decreased the risk of unknown migration of the NGT since the X-ray was taken. Nevertheless, using X-ray taken prior to the trial as the reference by which the performance of the device is compared can produce an error caused by the difference between the orientation of the X-ray source and the sensor array to the NGT. However, this error probably occurred randomly in both feasible and infeasible cases, thus should not bias the comparison. Moreover, in clinical practice, the trajectory of the NGT determined by the tracking device and by X-ray may be more relevant than the absolute difference between them.

Our results show that, in 5 out of 10 patients, the magnet tracking device feasibly detected the location of the NGT from the sternal angle to a level below the xiphisternum when the sensors were placed on the sternum (beginning from the sternal angle), but tracking was not feasible in most patients when the sensors were placed on the epigastrium (beginning from the xiphisternum). In the infeasible cases, the tracking either stopped abruptly or produced wide zig-zag lines indicating an unacceptable degree of error. Several reasons may explain this. First, motion of the chest wall during breathing can impair the performance of the device because the movement of the sensors with the chest wall is not accounted for in the tracking algorithm. Although we did not measure sensor movement directly, the sensors probably moved more when they were on the epigastrium than they were on the sternum because the sternum is relatively fixed in position during respiration compared to the ribs and the diaphragms, which in turn transmit the movement to the distensible abdominal wall. That a longer distance between the sternal angle and the tip of the xiphisternum, and higher body weight are observed in patients with poor tracking may also be explained by greater chest wall movement in patients with such body habitus, although this needs to be proven. Second, the magnet may have pitch angle larger than $5\pi /18$ in the nasal cavity and pharynx when tracking commenced, violating the preset search range of the algorithm^17^. This violation is likely to cause failure of tracking. Third, the magnet moved too distantly from the sensors as it descended into the stomach. This should decrease the tracking accuracy because of diminishing magnetic field strength. As Fig. 8 shows, the sagittal distance between the sensors and the NGT can be as long as nearly 15 cm posterior to the heart in a patient, greater than previous observations^22^.

This trial demonstrates that this magnet tracking system can locate the NGT at the bedside in the general ward of an urban hospital provided the device is properly sited. The prototype shows safety for clinical use, causes minimal discomfort, and can locate the NGT expeditiously. To develop it further for general clinical use, we must improve the accuracy, range of tracking, and prove its ability to detect the tip of the NGT which is most crucial in confirming placement. One proposed enhancement involves capturing the chest motion using the inertia measurement units and integrating this data into the algorithm to enhance the tracking results. Additionally, the preset search range can be dynamically adjusted to expand the volume of the search zone. Modifications to the number and arrangement of sensors will be explored to extend the tracking range and improve the tracking accuracy. Shielding for the sensors can be added to mitigate environmental EM noises, enabling its use in a wider range of clinical environments. Furthermore, the magnet with guidewire will be resized to make it compatible with most of the commercially available NGT and to eliminate the risk of extrusion from the side holes. Last but not least, the tracking procedures will be simplified for ease of use by most practitioners.

## Conclusion

This low-cost magnet tracking device can locate the Ryle’s tube at point-of-care in 50% of the patients in this first-in-human study. Further refinement is anticipated before validation in less controlled clinical settings.

### Supplementary Information

Supplementary Information.

## Data Availability

The datasets analysed during the current study are available from the corresponding author upon reasonable request.
