# Supplementary material for: Feasibility of a low-cost magnet tracking device in confirming nasogastric tube placement at point of care, a clinical trial
Source: Sci Rep. 2024 Mar 25;14:7068. doi: 10.1038/s41598-024-57455-7 (PMC10963729; doi:10.1038/s41598-024-57455-7)
Supplement: Supplementary file 1 — Supplementary Information. [file 41598_2024_57455_MOESM1_ESM.docx]

# Appendix A

# Data Processing for Comparison with Chest X-ray

### 1. Reconstruction of the trajectory of the nasogastric tube

To plot the trajectory of the magnet inserted into the nasogastric tube, only Z-values and respective Y-values are used. Figure A-1 plots the Z-value vs Y-value for Subject 08 with the upper sensor pair (USP) placed on the sternal angle. The colour bar on the right shows the time stamp.

For comparison with the measurements from chest X-ray (CXR), the data points near specific Z-values (from 0 cm to 20 cm at 2cm intervals) were extracted. The insertion and withdrawal were determined by the timestamp. The averaged values $(\bar{y}_{LSP,k},\bar{z}_{LSP,k})$ and $(\bar{y}_{USP,k},\bar{z}_{USP,k})$, $k=0,2,4,\ldots,20$, were calculated using the formulars below:


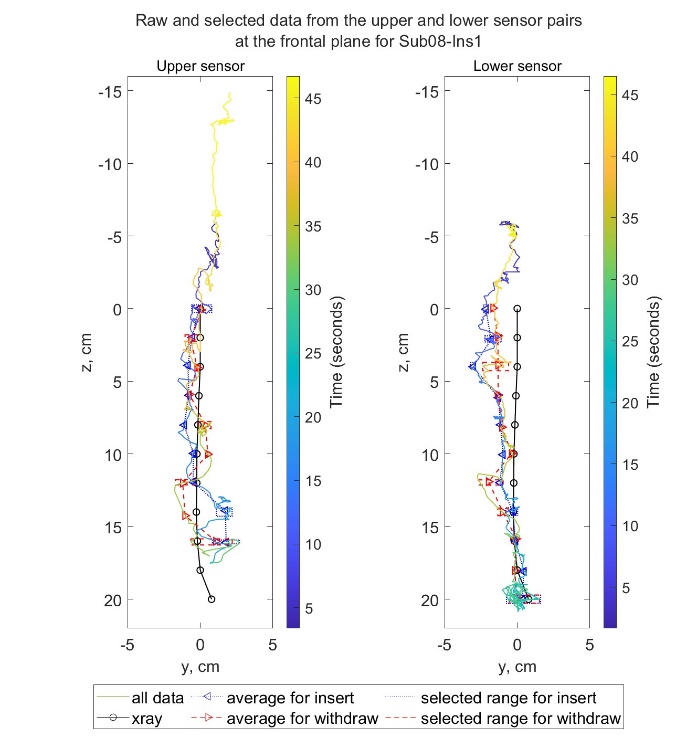


Figure A‑1: The lower sensor pair (LSP) and upper sensor pair (USP) measurements’ Z-value and Y-value for Subject 08 with the USP placed on the sternal angle. The chest X-ray measurements are plotted as solid black circles.

| $\begin{matrix} {(\bar{y},\bar{z})}_{LSP,k}=\sum{(y,z)}_{LSP,i}/n & if \left\vert z_{LSP,i}-k \right\vert\leq0.3 cm \end{matrix}$ | (1) |
| --- | --- |
| $\begin{matrix} {(\bar{y},\bar{z})}_{USP,k}=\sum{(y,z)}_{USP,j}/n & if \left\vert z_{USP,j}-k \right\vert\leq0.3 cm \end{matrix}$ | (2) |

where $n$ is the number of points that satifies the condition and LSP denotes lower sensor pair.

$(\bar{y}_{USP,k},\bar{z}_{USP,k})$ and $(\bar{y}_{LSP,k},\bar{z}_{LSP,k})$ are plotted in Figure A-2 (a) and (b).

These results were merged according to formula *(3)*.

| ${(x,y,z)}_{merged}= \left\{ \begin{aligned} \begin{matrix} {(x,y,z)}_{LSP} & z_{LSP}\geq6 cm \end{matrix} \\ \begin{matrix} {(x,y,z)}_{USP} & z_{USP} <6 cm \end{matrix} \end{aligned} \right.$ | (3) |
| --- | --- |

The merged output, $(\bar{y}_{merged,k},\bar{z}_{merged,k})$, is plotted in Figure A-2 (c).


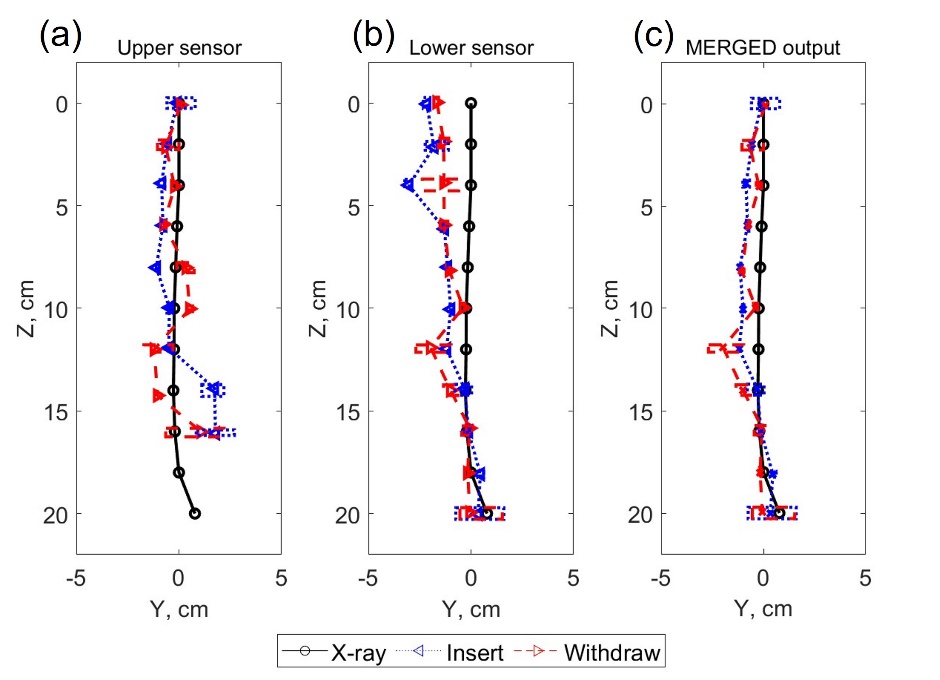


Figure A‑2 averaged values of selected processed data from upper sensor pair (a) and lower sensor pair (b). The merged averaged values are presented in (c).

Compared with the CXR measurements, their average absolute differences in the Y-axis are defined as follows:

- $\mu_{y,ic}$ between merged data and CXR measurements during insertion

| $\mu_{y,ic}=\sum\left\vert\bar{y}_{merged,insert,k}-y_{CXR,k} \right\vert/n_{ic}$ | (4) |
| --- | --- |

- $\mu_{y,wc}$ between merged data and CXR measurements during withdrawal

| $\mu_{y,wc}=\sum\left\vert\bar{y}_{merged,withdraw,k}-y_{CXR,k} \right\vert/n_{wc}$ | (5) |
| --- | --- |

- $\mu_{y,iw}$ between merged data during withdrawal and during insertion

| $\mu_{y,iw}=\sum\left\vert\bar{y}_{merged,withdraw,k}-\bar{y}_{merged,insert,k} \right\vert/n_{iw}$ | (6) |
| --- | --- |

where, $n_{ic}$, $n_{wc}$, and $n_{iw}$ are numbers of available points within 0 to 20 cm range in each case.

Moreover, the box around the point in Figure A-2 indicates the distribution range of the tracked data. A smaller box size means the tracking process is more stable and no box means only one data point.

From the physical measurement of Subject 8, the xiphisternum is 10 cm away from the 2^nd^ intercostal space. Thus, connecting the 6^th^ point and last point, the gradient of the line inferior to the xiphisternum is positive, suggesting that a left-turn of the guidewire exists below the xiphisternum.

### 2. Pre-processed Data from All Participants Who Completed The Trial

Here, we provide the sensor readout obtained in real time as the magnet was inserted or withdrawn from the nasogastric tubes of the research participants (Fig. A-3 and Fig. A-4).

**Figure A-3:** pre-processed location of the nasogastric tube determined by the magnet tracking device when the upper sensors are placed on the sternal angle


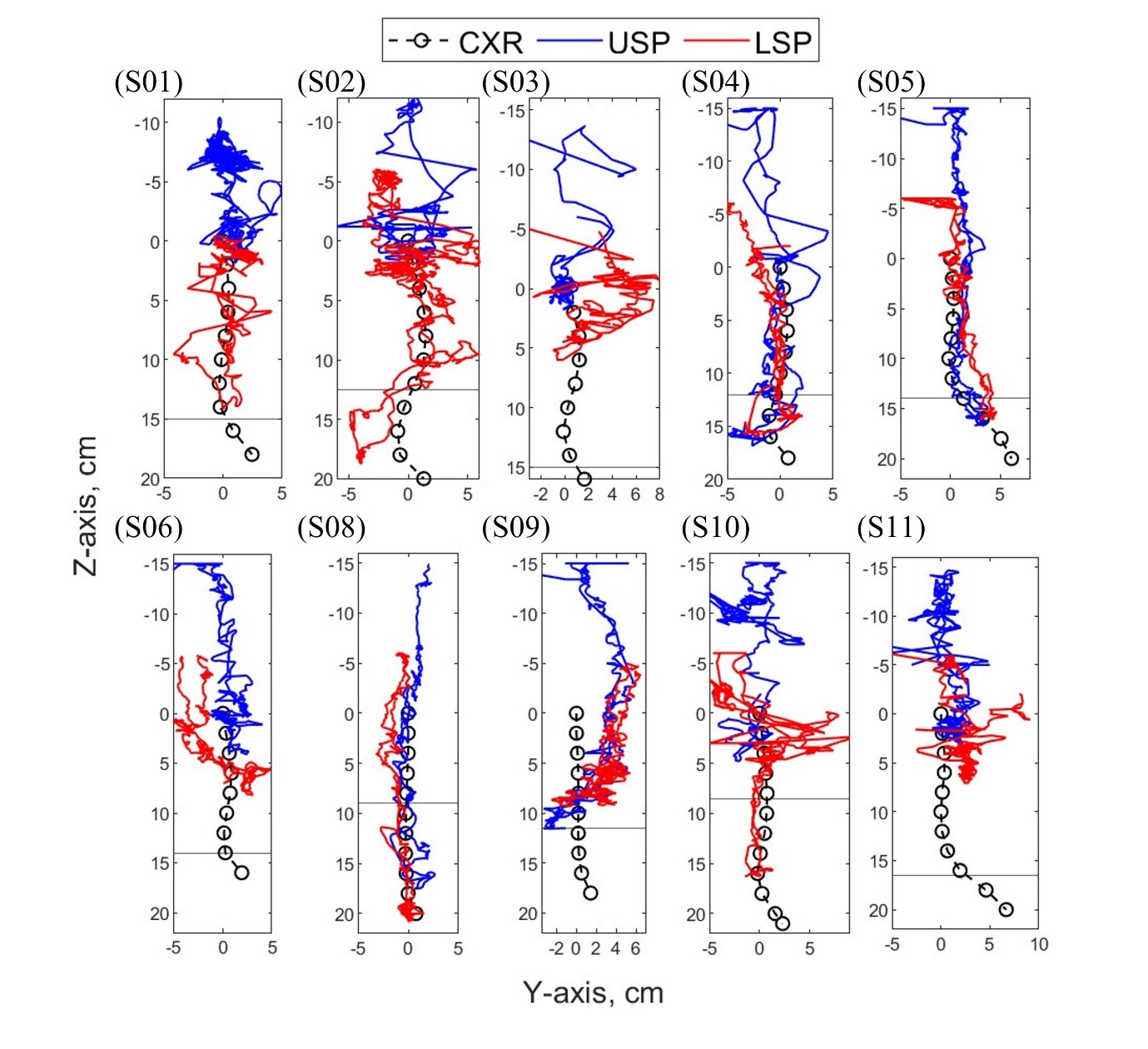


**Figure A-4**: pre-processed location of the nasogastric tube determined by the magnet tracking device when the upper sensors are placed on the xiphisternum


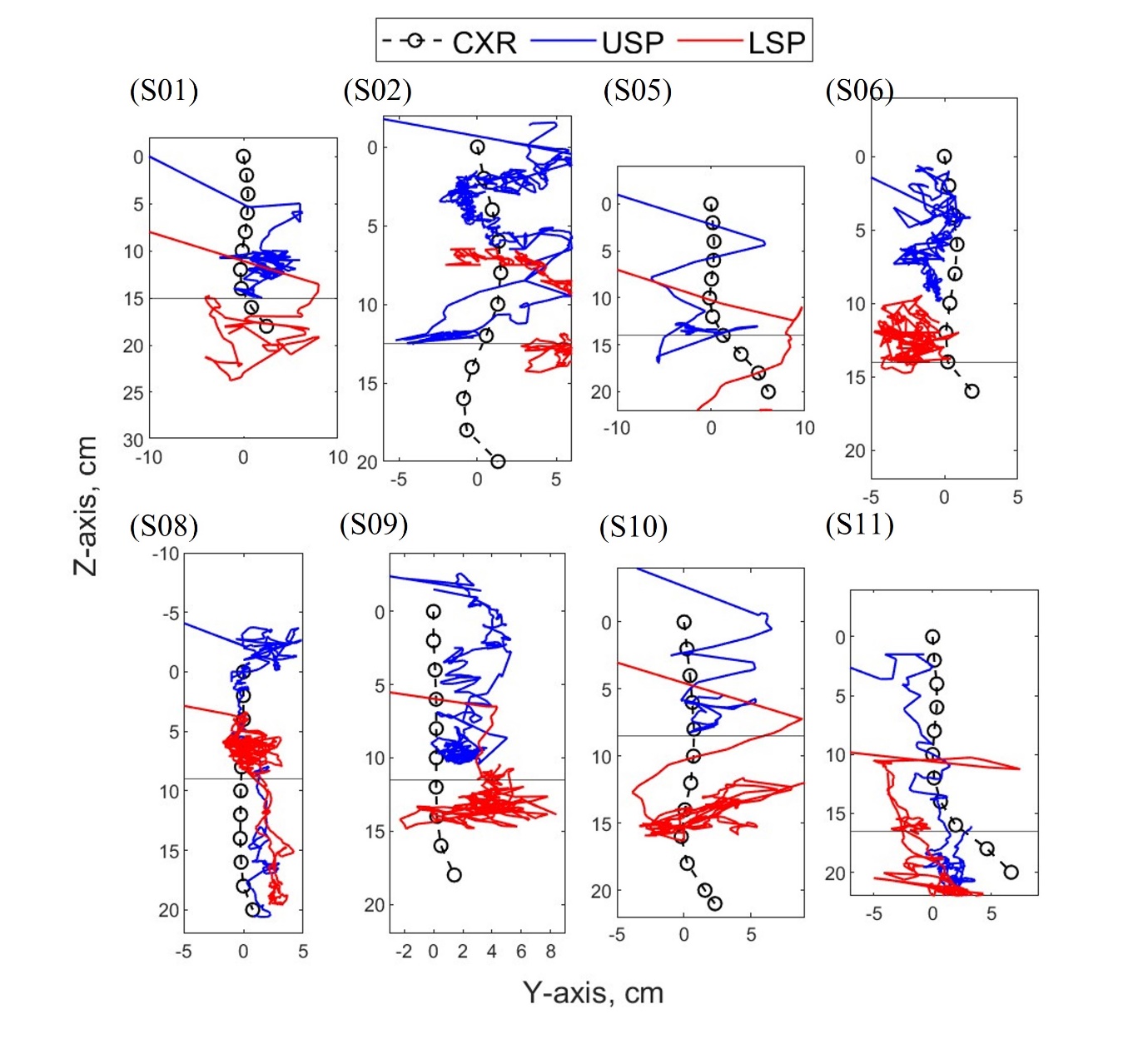


**Legend**

Each graph shows data from an individual participant labeled by the subject number. The black circles, spaced 2cm apart, denote the location of the nasogastric tube seen on chest X-ray. CXR: chest X-ray; USP: upper sensor pair; LSP: lower sensor pair; Z-axis represents the vertical distance from the upper sensor pair with positive values indicating movement downwards from the sensors and negative values indicating movement upwards from the sensors; Y-axis represents horizontal deviation from the vertical reference line down the midpoint of the sternum at the level of the 2^nd^ intercostal space , with positive values indicating deviation to the left and negative values indicating deviation to the right; horizontal black line indicates the level of the xiphisternum

### Appendix B

### Comparing The Tracking Results Between The Upper and Lower Sensor Pairs

As the measurements from LSP and USP are recorded separately, they can be displayed and compared (**Figure B-1)**. Because the measurements from LSP and USP have overlaps, the magnet can be detected by both sensor pairs during the tracking. In this overlapped region, the average differences of x-value, y-value, and z-values between the LSP and USP, $\mu_{xyz}$, can be computed using (7).

| $\mu_{xyz}=(\frac{\sum\left\vert x_{LSP,i}-x_{USP,j} \right\vert}{n}+\frac{\sum\left\vert y_{LSP,i}-y_{USP,j} \right\vert}{n}+\frac{\sum\left\vert z_{LSP,i}-z_{USP,j} \right\vert}{n})/3$ | (7) |
| --- | --- |

where, $x_{LSP,i}$, $y_{LSP,i}$, $z_{LSP,i}$ are the positions of i-th LSP tracked point; $x_{USP,j}$, $y_{USP,j}$, $z_{USP,j}$ are the positions of j-th USP tracked point. The i-th LSP tracked point and j-th USP tracked point share a nearly identical time stamp.

The measurements also have a range in the x-axis, y-axis, and z-axis, akin to the sagittal, frontal and longitudinal axis of the X-ray. The range of values for the x and y axes is smaller range than that of the z-axis because of the anatomical location of the nasogastric tube. For successful localisation of the nasogastric tube, the z range is crucial, and is calculated as follows.

| $Z_{range}=\max\left( z_{LSP,i},z_{USP,j} \right)-max(0, \min\left( z_{LSP,i},z_{USP,j} \right))$ | (8) |
| --- | --- |


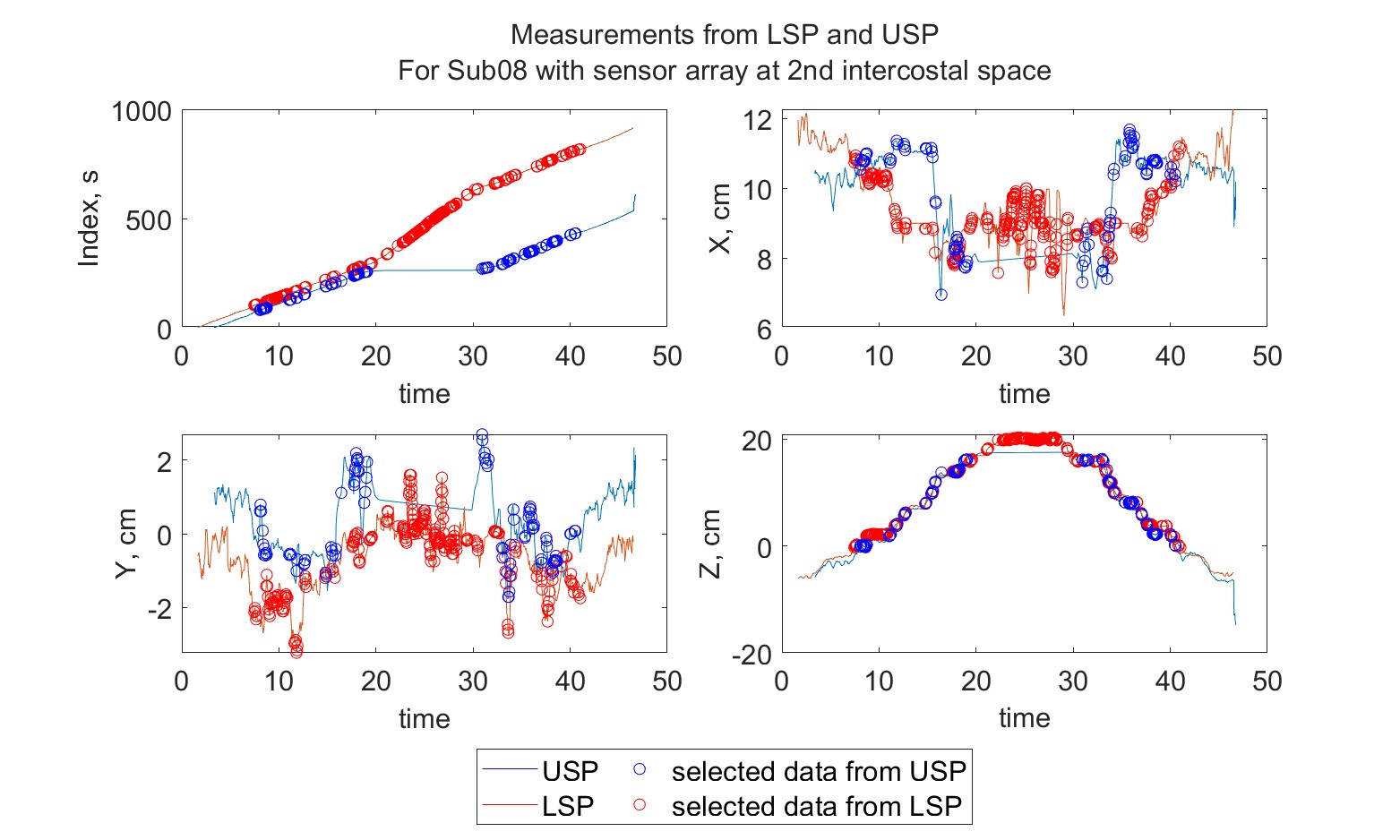


Figure B-1: The processed data’s index, X-value, Y-value, and Z-value versus time.
